# Confidence–gradient reweighting and lightweight feature enhancement algorithm for steel surface defect detection

**DOI:** 10.1038/s41598-026-36543-w

**Published:** 2026-01-18

**Authors:** Linxuan Chen, Cunhan Guo, Xiaofang Wu, Huilin Xu, Shuangmei Chen, Junwu Lin

**Affiliations:** 1 College of Intelligent Manufacturing, Putian University, Putian, 351100 China; 2 School of Computer Science and Technology, Beijing Institute of Technology, Beijing, 100081 China; 3 College of Artificial Intelligence, Putian University, Putian, 351100 China; 4 Fujian Putian Licheng Paper Industry Co., Ltd., Jiaoxi Village, Huating Town, Chengxiang District, Putian, 351100 China

**Keywords:** Steel surface defect detection, Small object detection, Class imbalance, YOLO, Long-tailed distribution, Engineering, Mathematics and computing

## Abstract

Steel surface defect detection is susceptible to small target sizes, low contrast, and class imbalance. To this end, we propose the Gradient-Reweighting with Awareness of Confidence and Lightweight Feature Enhancement (GRACE) algorithm built upon YOLO11s, composed of two synergistic modules: Dynamic Sampling with Confidence-Gradient Balanced Sampling Mechanism (DS-CBSM++) performs dynamic reweighting via joint confidence-gradient feedback, improving the separability of hard examples and long-tailed classes; Lightweight Feature Enhancement Network (Lite-FEN) introduces lightweight channel/spatial enhancement at the P3 layer to strengthen shallow textures and boundary cues while keeping computation low. Experiments on the NEU-DET dataset show that the baseline YOLO11s achieves an mAP@0.5:0.95 of 42.66% and an mAP@0.5 of 74.69%. GRACE achieves 43.66% and 75.88%, respectively, improving over the baseline by 1.00 percentage points and 1.19 percentage points, with 9.56 M parameters, suitable for real-time inference. These results indicate that GRACE yields more robust detection and localization of small defects under complex textured backgrounds.Additional experiments on the GC10-DET and X-SDD datasets further confirm that GRACE maintains competitive performance across different steel surface defect distributions.

## Introduction

Quality control in steel and electronics manufacturing relies heavily on surface defect inspection, directly affecting product quality and line yield^1–3^. In practice, defects are often small in size, irregular in shape, and embedded in complex background textures; manual inspection is highly subjective and inefficient, and automated optical inspection (AOI), though widely deployed, remains prone to false positives and false negatives under weak defect contrast and complex textures, making it difficult to robustly cover diverse fine-grained defect types. Consequently, academia and industry have shifted toward vision-centric automated inspection systems and have established public datasets to support benchmarking; among them, the NEU-DET dataset contains six common surface defects of hot-rolled steel strips^4,5^—inclusion, patches, crazing, rolled-in_scale, scratches, and pitted surface—providing a representative evaluation platform for small-object and weak-texture defect detection.

Early methods largely relied on handcrafted features and traditional classifiers—for example, texture- or edge-based descriptors combined with thresholding or shallow learners for recognition; such schemes are inexpensive and easy to deploy, but they are sensitive to imaging conditions, noise, and parameter settings, with limited cross-domain generalization. Subsequent machine-learning-based automated visual inspection (AVI) alleviated subjectivity and efficiency issues to some extent and can achieve favorable detection speed and accuracy under standardized illumination and backgrounds. However, when defects exhibit low contrast, fine-grained textures or diverse morphologies, handcrafted features with fixed representational capacity still struggle to capture discriminative information; the models are brittle under data distribution shift and entail higher maintenance overhead and iteration costs.

With the rise of deep learning, end-to-end convolutional networks have markedly improved representational capacity and robustness in industrial vision, with segmentation, classification, and detection advancing in parallel; in steel strip/plate surface-defect scenarios, many studies show that detector-centric pipelines can deliver higher detection and localization accuracy while maintaining inference efficiency. With the development of generic techniques such as feature pyramids, multi-scale fusion, and hard-example reweighting, detectors further enhance the visibility and localization stability of small objects in complex backgrounds, yet challenges persist under long-tailed categories, small pixel footprints, and texture-similarity interference^6^. For real-time monitoring—especially under the real-world production constraints on latency and model light-weighting^7,8^—balancing accuracy and efficiency remains a central research focus. More broadly, deep learning-based models have recently shown strong performance in diverse application domains, including microscopic biomedical image super-resolution, mechanical transmission modelling, financial portfolio optimisation, and multimodal zero-shot learning^9–12^, underscoring their versatility for complex, data-intensive tasks.

However, existing deep-learning-based steel surface defect detection methods still exhibit several limitations in practical deployment. First, many works that address long-tailed distributions and hard examples rely on fixed loss re-weighting or sampling schemes (e.g., focal loss and class-balanced loss)^13,14^, which mainly exploit static statistics such as class frequency while providing little adaptive feedback from the evolving confidence or gradient signals during training; as a result, the learning effect for minority classes and low-contrast defects remains limited. Second, to strengthen the representation of fine-grained textures and weak boundaries, numerous methods directly stack channel/spatial attention modules (e.g., SE and CBAM)^15,16^ or introduce more complex large-kernel and multi-branch structures into the backbone or feature pyramid. Although these designs often improve accuracy on benchmark datasets, they also substantially increase the number of parameters and computational cost, which makes deployment on resource-constrained, real-time production lines difficult. Finally, many steel surface studies report results only on one or two datasets (e.g., NEU-DET or GC10-DET)^17^ and lack systematic cross-dataset evaluation under different steel grades, process conditions, and background textures, so the robustness of the algorithms in real manufacturing environments is not fully verified. Motivated by these limitations, the GRACE framework in this paper aims to compensate for the above issues from the perspectives of dynamic sampling and lightweight feature enhancement, and provides unified, reproducible empirical analysis on multiple steel surface defect datasets.

Despite advances in sampling strategies, loss design, and attention modeling, scenarios with small-scale, low-contrast, irregular steel-surface defects and long-tailed categories^18^ still exhibit pronounced false positives and false negatives, and localization is readily disturbed by background textures. To address these issues, we build the modular, small-object-aware GRACE algorithm on YOLO11s, centered on the co-optimization of a data-driven training strategy and in-model feature enhancement, balancing accuracy, efficiency, and deployability, with systematic validation on the NEU-DET dataset^19^.

Our contributions are as follows:

1. We propose the Gradient-Reweighting with Awareness of Confidence and Lightweight Feature Enhancement (GRACE) algorithm, which prioritizes training and representational resources for long-tailed, low-contrast small-object classes; while the overall mAP increases, a few high-frequency classes exhibit a slight decline—a controllable trade-off aligned with hard-example optimization;
2. We introduce Dynamic Sampling with Confidence-Gradient Balanced Sampling Mechanism (DS-CBSM++). It introduces dual feedback from forward confidence and backward gradients during data loading and training, performing class- and difficulty-aware dynamic reweighting and in-batch composition to suppress easy-sample dominance and majority-class gradient bias, thereby improving coverage and convergence stability for tail classes and hard samples. The mechanism interfaces seamlessly with existing training pipelines;
3. We design Lightweight Feature Enhancement Network (Lite-FEN). At the early feature layer (P3), it embeds dual-path channel/spatial lightweight attention and boundary-friendly filtering, refining texture and edge representations, strengthening small-object separability, while keeping the parameter budget controlled and suitable for real-time detection and industrial deployment requirements.

The remainder of this article first reviews related work; then presents the results and analysis (including setup, comparisons, ablation studies, and visualizations); followed by a discussion; and finally describes the methods, including the dataset, baseline, evaluation metrics, and implementation details. Required statements (data availability, author contributions, and competing interests) appear at the end.

## Related work

### Evolution of industrial defect detection methods

Industrial surface defect inspection has transitioned from manual inspection and rule-based AOI to learning-driven, deep-learning-integrated frameworks^20^, a shift that coincides with the growing prominence of class imbalance and multi-scale challenges in industrial settings. Early approaches relied on thresholding, edge- or texture-based operators and descriptors, and template matching, together with handcrafted features such as LBP, GLCM, and HOG and shallow classifiers; this route is low-cost and interpretable, yet sensitive to illumination, process drift, and misalignment, with limited generalization across devices and production batches, and it struggles to robustly cover low-contrast and small-scale defects. With the advent of deep learning and end-to-end feature learning, segmentation, classification, and detection advanced in parallel; the detection paradigm evolved from preprocessing-plus-classifier to dense-prediction architectures that integrate multi-scale features with regression. In production practice, pipelines that first generate candidate regions and then classify/regress within them have matured, while approaches that directly regress classes and boxes on multi-scale feature maps have also been refined, typically paired with feature pyramids for scale variation and small objects; two-stage methods (e.g., Faster R-CNN) excel in proposal quality and localization accuracy but incur higher latency and resource usage; single-stage methods such as YOLO, SSD^21,22^ achieve high-throughput inference and good deployability via anchor-based/anchor-free designs, feature pyramids, and lightweight backbones, and thus have become mainstream for real-time industrial scenarios. In this context, research focus has shifted toward improving small-object visibility, learning from long-tailed data, and efficient modeling of shallow details, laying the groundwork for subsequent co-optimization on the training side and the feature side.

### The YOLO series and lightweight development

Since YOLOv1 introduced the single-stage unified regression paradigm, object detection has shifted from the two-stage “proposal generation–classification refinement” pipeline to end-to-end high-speed inference^23^. YOLOv2 balanced accuracy and speed through anchor boxes, batch normalization, and multi-scale training; YOLOv3 achieved cross-scale fusion via a residual backbone and feature pyramids; YOLOv8 introduced an anchor-free mechanism that removes anchor dependence, reduces manual prior bias, and improves adaptability to multi-scale detection^24,25^. Subsequent versions have continued engineering iterations on backbone factorization, path aggregation, data augmentation, and label assignment, with compact variants progressively adapted to edge devices. Recent advances emphasize anchor-free prediction, decoupled detection heads, and lightweight fusion structures, reducing prior dependence and computational cost while improving small-object visibility and bounding-box regression stability. As a representative, YOLO11^26^ carries these strengths forward, enhancing fine-grained representations at early scales while controlling latency and memory footprint, making it well suited for industrial real-time detection. The series’ modular and lightweight evolution^27–29^ also provides favorable interfaces for training-side dynamic reweighting and shallow feature-enhancement methods. Balancing performance and efficiency, and following the official n/s/m/l/x variants, we adopt YOLO11s as the baseline model in subsequent experiments.

### Small-object detection and class-imbalance handling

In industrial scenarios, steel-surface defects are predominantly small objects, the instance-level class distribution is markedly long-tailed, the imbalance between small-object and foreground–background pixels further increases false-positive and false-negative risks, and background clutter is severe^30^. Although YOLO11s has limited capacity for small-object detection, two challenges remain: (i) the scarcity of samples in long-tailed categories leads to high miss rates for minority classes; (ii) shallow texture and boundary cues are progressively attenuated through deep feature fusion, weakening discriminative representations.

In recent years, many studies have approached the problem from the architectural side, improving detection performance and robustness through optimizations of network architectures and path designs. To reduce latency and computational cost, work has continued on lightweighting and backbone refactoring: CRFD-YOLO achieves hundred-fps real-time performance on NEU-DET while maintaining accuracy, and SS-YOLO replaces the backbone with MobileNetV3 to cut parameters and accelerate inference^31,32^. Global attention and cascaded fusion enhance discrimination of complex textures and fine-grained defects; GC-Net and GCF-Net^33^ report stable gains on steel-surface datasets. Transformers and sparse attention strengthen global–local coupling, and DSAT^34^ has been validated across multiple datasets. For small-area defects, SRN-YOLO^35^ improves separability via super-resolution and multi-scale reconstruction. Overall, these methods act mainly on the inference architecture; class-imbalance handling and hard-example modeling during training remain comparatively underdeveloped.

The two modules proposed in this work, DS-CBSM++ and Lite-FEN, target the training-sampling stage and the shallow-feature stage, respectively, establishing a modular, plug-and-play mechanism that effectively balances small-object sensitivity and lightweight inference efficiency. As a composite implementation of reweighting and lightweight feature enhancement, the strategy achieves coordinated improvements in the training pipeline and inference capability.

## Results and analysis

### Experiment setting

The proposed model is built on the PyTorch deep-learning framework and the Ultralytics YOLO library. The hardware configuration includes an NVIDIA GeForce RTX 3070 Ti GPU with 8 GB of VRAM and the Windows 11 operating system. The software stack comprises Python 3.8, PyTorch 2.0.1+cu118, and torchvision 0.15.2+cu118. Training hyperparameters are set as follows: 200 total epochs; batch size = 16; input size = 640; initial learning rate = 0.001; and an SGD optimizer for stable convergence.

Evaluation on the NEU-DET dataset uses five metrics—mAP@0.5:0.95, mAP@0.5, Precision, Recall, and F1—as given by equations (1)–(5). The definitions of these metrics are as follows:

1 $$\begin{aligned} & \textrm{mAP}@0.5\!:\!0.95 = \frac{1}{10}\sum _{\textrm{IoU}=0.5}^{0.95} \textrm{AP}_{\textrm{IoU}}. \end{aligned}$$

2 $$\begin{aligned} & \textrm{mAP}@0.5 = \frac{1}{N}\sum _{i=1}^{N} \textrm{AP}_i. \end{aligned}$$

3 $$\begin{aligned} & \textrm{Precision} = \frac{\textrm{TP}}{\textrm{TP} + \textrm{FP}}. \end{aligned}$$

4 $$\begin{aligned} & \textrm{Recall} = \frac{\textrm{TP}}{\textrm{TP} + \textrm{FN}}. \end{aligned}$$

5 $$\begin{aligned} & \textrm{F1} = \frac{2\,\textrm{Precision}\cdot \textrm{Recall}}{\textrm{Precision}+\textrm{Recall}}. \end{aligned}$$

In equations (1)–(5), the symbols are defined as follows. In equation (1), $\textrm{IoU}$ denotes the intersection-over-union between a predicted bounding box and its corresponding ground-truth box, and $\textrm{AP}_{\textrm{IoU}}$ is the class-averaged average precision computed at a fixed IoU threshold. We follow the COCO protocol and average $\textrm{AP}_{\textrm{IoU}}$ over ten IoU thresholds from 0.50 to 0.95 with a step of 0.05 to obtain $\textrm{mAP}@0.5\!:\!0.95$. In equation (2), *N* is the number of defect classes and $\textrm{AP}_i$ is the average precision of class *i* at IoU = 0.50; $\textrm{mAP}@0.5$ is the mean of $\textrm{AP}_i$ over all *N* classes. In equations (3) and (4), $\textrm{TP}$, $\textrm{FP}$ and $\textrm{FN}$ denote the numbers of true positives, false positives and false negatives at the chosen confidence threshold: a detection that matches a ground-truth box of the same class with IoU above the threshold is counted as a true positive, an unmatched detection as a false positive, and an unmatched ground-truth box as a false negative; background regions without annotations are ignored. Finally, in equation (5), Precision and Recall are combined into the F1 score, i.e., the harmonic mean of Precision and Recall, which is computed at the dataset level from the above counts.

### Overall performance on NEU-DET

Under the same split, training strategy, and input size on NEU-DET, we compare YOLOv5s, YOLOv8s, YOLO11s, RT-DETR-L, and Faster R-CNN (R50-FPN). Table 1 summarizes the results.

**Table 1:** Performance comparison of different object detection models.

Model	mAP@0.5: 0.95 (%)	mAP@0.5 (%)	P (%)	R (%)	F1 (%)	Param (M)	Latency (ms/img)
YOLOv5s	42.51	73.24	73.15	69.82	71.45	**7.20**	14.11
YOLOv8s	39.97	72.14	**74.57**	66.98	70.57	11.16	**12.52**
YOLO11s	42.66	74.69	70.72	**73.66**	72.16	9.44	15.97
RT-DETR-L	37.52	68.97	69.42	64.14	66.68	32.00	43.47
Faster R-CNN (R50-FPN)	33.82	66.25	59.49	71.19	64.82	43.71	30.10
**(GRACE) Ours**	**43.66**	**75.88**	73.62	72.33	**72.97**	9.56	16.93

GRACE attains the best performance on two core metrics, with mAP@0.5:0.95 = 43.66% and mAP@0.5 = 75.88%; its F1 score reaches 72.97%, an improvement of 0.81 percentage points over the next-best YOLO11s, indicating a balanced trade-off between precision and recall while maintaining high mAP. The parameter count is 9.56 M, comparable to that of YOLO11s (9.44 M), and the measured inference latency is 16.93 ms per $640\times 640$ image (59.07 FPS), only slightly higher than YOLO11s (15.97 ms, 62.62 FPS).

We use YOLO11s as the primary comparator, with YOLOv8s and YOLOv5s as references. YOLOv8s shows higher Precision but lower Recall, whereas YOLOv5s is relatively balanced. Relative to YOLO11s, GRACE trained on NEU-DET improves mAP@0.5:0.95 and mAP@0.5 by 1.00 percentage points and 1.19 percentage points, respectively, and increases F1 by 0.81 percentage points; compared with YOLOv8s, at comparable Precision it provides more stable Recall, which is more favorable for low-contrast and small-scale defects. Overall, the gains center on the trade-off between false-positive control and stable detection, which is non-trivial given that GRACE keeps almost the same parameter count and training configuration as the YOLO11s baseline.

Two-stage and Transformer-based detectors show no clear advantage on this dataset. RT-DETR-L attains mAP@0.5:0.95 of 37.52%, mAP@0.5 of 68.97%, and F1 of 66.68%, with 32.00 M parameters; Faster R-CNN (R50-FPN) reports mAP@0.5:0.95 of 33.82% and mAP@0.5 of 66.25%, with 43.71 M parameters, incurring substantially higher compute and memory overhead. Consistently, their inference latencies (43.47 ms and 30.10 ms per image, respectively) are much higher than those of the one-stage detectors, reinforcing the advantage of a lightweight design for real-time inspection. Given the NEU-DET instance-size statistics—dominated by medium-scale instances, followed by large ones, with relatively few small instances—a lightweight one-stage framework offers a better accuracy–efficiency trade-off.

**Fig. 1:**
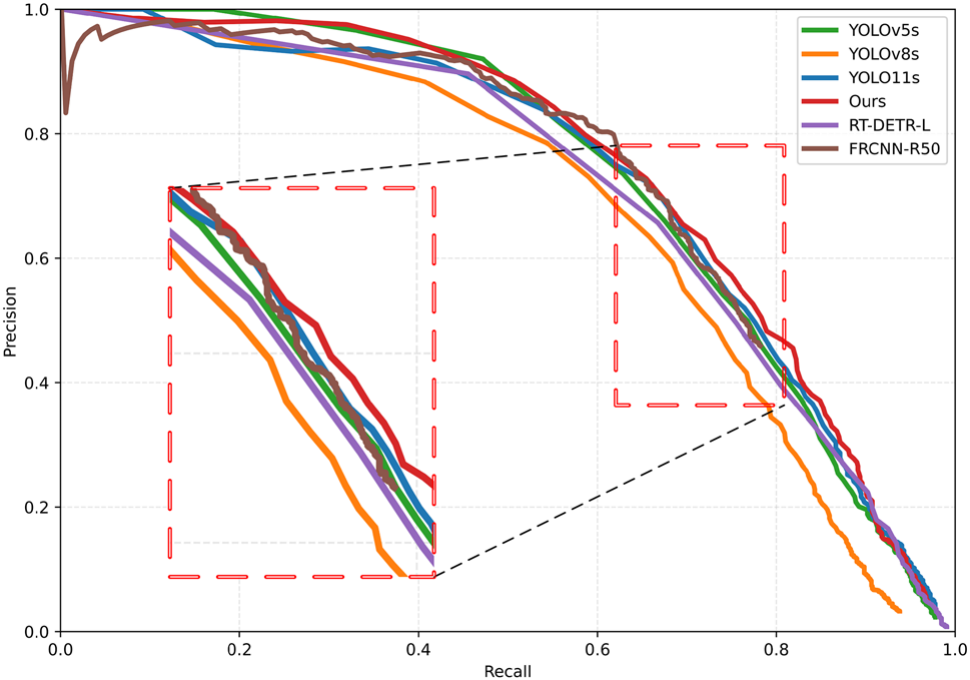
PR curves of multiple models (IoU = 0.50). The proposed method outperforms the baselines over the recall range of approximately 0.63–0.85 while remaining comparable at the high-confidence end, reflecting a better precision–recall trade-off.

**Fig. 2:**
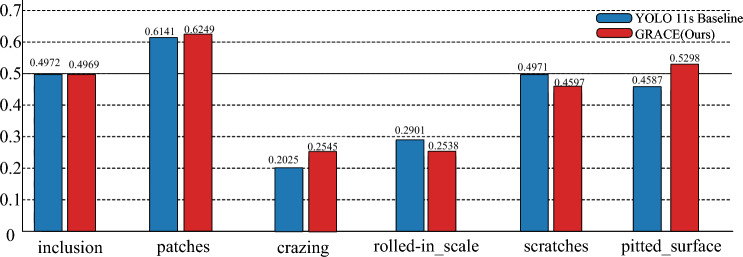
Per-class AP (AP@0.5:0.95) on NEU-DET. Compared with YOLO11s, GRACE yields pronounced gains on crazing and pitted_surface, a slight gain on patches, near parity on inclusion, and slight drops on rolled-in_scale and scratches; overall, mAP@0.5:0.95 shows a net increase.

To further characterize overall detection reliability and class-wise gains, Fig. 1 shows the PR curves of multiple models at IoU = 0.50, and Fig. 2 reports per-class AP for the six defect categories. GRACE maintains higher Precision over the main operating range of recall approximately 0.63–0.85, and it nearly overlaps the baselines at the high-confidence end, indicating that the quality of high-confidence predictions is not compromised. Aggregating the metrics in Table 1 (reported as relative changes from YOLO11s baseline), mAP@0.5:0.95 and mAP@0.5 increase by 2.34% and 1.60%, respectively; Precision rises by 4.10%, while Recall decreases slightly by $1.81\%$.

Class-wise, AP gains are pronounced for crazing (up 5.20 percentage points) and pitted_surface (up 7.11 percentage points); patches rises slightly (up 1.08 percentage points); inclusion is essentially flat (down 0.03 percentage points). The rolled-in_scale and scratches drop by 3.63 percentage points and 3.74 percentage points, respectively. This pattern aligns with the design: DS-CBSM++ improves coverage of minority and low-confidence samples, while Lite-FEN enhances shallow textures and weak-contrast details, increasing sensitivity to fine textures and dim defects. Overall, with minimal additional inference overhead, the combined strategy yields a better precision–recall trade-off and a higher aggregate mAP.

### Cross-dataset generalization on GC10-DET and X-SDD

To further examine whether GRACE overfits NEU-DET, we conduct cross-dataset generalization experiments on the two steel surface defect datasets GC10-DET^17^ and X-SDD^36^ introduced above. In both cases, we compare only the baseline YOLO11s and our GRACE, reusing the same training schedule and hyperparameters as on NEU-DET without any dataset-specific tuning, so as to isolate the effect of the proposed architectural changes.

On GC10-DET, mAP@0.5:0.95 increases from 29.02% to 32.38% (up 3.36 percentage points), and mAP@0.5 from 58.66% to 61.14% (up 2.48 percentage points); Recall increases from 61.65% to 63.68% (up 2.03 percentage points), while Precision slightly decreases from 56.84% to 55.47% (down 1.37 percentage points). The corresponding F1 score, computed as the harmonic mean of Precision and Recall, increases slightly from 59.15% to 59.29% (up 0.14 percentage points), indicating a near-neutral change in the overall precision–recall balance. Overall, GRACE recovers more defect instances on this benchmark and yields a more pronounced improvement in overall localization quality.

On X-SDD, where defect scales and background textures are more diverse, Precision increases from 66.94% to 74.68% (up 7.74 percentage points) and Recall from 56.71% to 60.89% (up 4.18 percentage points). Accordingly, the F1 score increases from 61.40% to 67.08% (up 5.68 percentage points), suggesting a more favorable precision–recall balance. mAP@0.5 increases marginally from 64.84% to 65.14% (up 0.30 percentage points), whereas mAP@0.5:0.95 decreases from 36.19% to 33.03% (down 3.16 percentage points), reflecting a localization trade-off under stricter IoU thresholds. Taken together, the two additional datasets show that GRACE maintains competitive performance across different steel surface defect distributions, rather than being tailored to the specific characteristics of NEU-DET alone. Representative Eigen-CAM visualizations on GC10-DET and X-SDD are provided in Supplementary Fig. S1.

### Ablation studies

To further validate the effectiveness of GRACE, we perform an ablation study by isolating DS-CBSM++ and Lite-FEN under identical experimental conditions, adopting a unified COCO evaluation protocol across all models. Table 2 summarizes the results.

**Table 2:** Ablation results on NEU-DET under a unified COCO evaluation protocol.

Model	mAP@0.5: 0.95 (%)	mAP@0.5 (%)	P (%)	R 1 (%)	F1 (%)	Param (M)
YOLO11s	42.66	74.69	70.72	**73.66**	72.16	**9.44**
YOLO11s + DS-CBSM++	43.69	75.95	72.77	72.59	72.68	**9.44**
YOLO11s + Lite-FEN	**45.14**	**76.25**	68.66	72.71	70.63	9.56
**GRACE (Ours)**	43.66	75.88	**73.62**	72.33	**72.97**	9.56

With DS-CBSM++ only, mAP@0.5:0.95 increases from 42.66% to 43.69% (up 1.03 percentage points), mAP@0.5 from 74.69% to 75.95% (up 1.26 percentage points); Precision rises to 72.77% (up 2.05 percentage points), Recall dips slightly to 72.59% (down 1.07 percentage points), and F1 improves to 72.68% (up 0.52 percentage points). With Lite-FEN only—which strengthens shallow textures and small-object boundaries—mAP@0.5:0.95 reaches 45.14% (up 2.48 percentage points) and mAP@0.5 reaches 76.25% (up 1.56 percentage points), the best among the ablations; however, Precision drops to 68.66% (down 2.06 percentage points), indicating more false positives.

Combining the two yields GRACE (Ours). With only a slight increase in parameter count (from 9.44 M for YOLO11s and YOLO11s + DS-CBSM++ to 9.56 M for YOLO11s + Lite-FEN and GRACE), Precision = 73.62% and F1 = 72.97% are the best in the group; the two mAP metrics remain high (43.66% for mAP@0.5:0.95 and 75.88% for mAP@0.5), comparable to the single-module optima. This indicates that distribution rebalancing on the sampling side (DS-CBSM++) and fine-grained enhancement on the feature side (Lite-FEN) are complementary: the former suppresses enhancement-induced false positives, while the latter mitigates missed detections of small, low-contrast defects that sampling alone cannot address. Overall, GRACE delivers a more stable precision–recall trade-off in the main operating range of industrial quality inspection and greater practical utility. All ablated variants share exactly the same YOLO11s backbone, detection head and training settings; only the presence or absence of DS-CBSM++ and Lite-FEN is changed, so the observed performance differences can be attributed to the proposed modules rather than to the underlying framework.

### Visualizations and case analysis

**Fig. 3:**
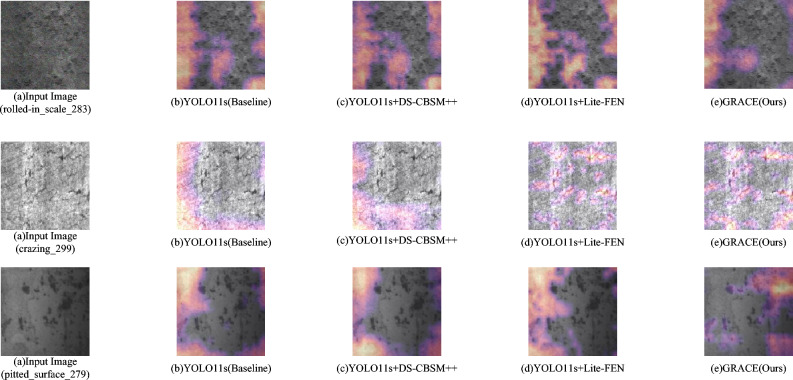
Eigen-CAM visualizations. (**a**) Input image; (**b**) YOLO11s; (**c**) YOLO11s + DS-CBSM++; (**d**) YOLO11s + Lite-FEN; (**e**) GRACE (Ours). All heatmaps use global min–max normalization with identical colormap and transparency, and are visualized from the same target layer.

Eigen-CAM produces class activation maps by extracting principal components of feature maps and does not rely on back-propagated gradients, making it suitable for robust visualization under low-contrast settings^37^. Figure 3 presents three NEU-DET samples—rolled-in_scale_283, crazing_299, and pitted_surface_279—whose textures are coarse, contrast is low, and background boundaries are blurry, which are representative. The heatmaps are uniformly normalized; yellow/orange denote high response, and purple/dark denote low response. Using rolled-in_scale_283 as an example: in (b), YOLO11s yields a diffuse response with spurious highlights in non-defect background, obscuring the target; in (c), adding DS-CBSM++ concentrates attention on the true defect and increases activation on small targets, indicating that this module effectively guides the model toward difficult samples; in (d), introducing Lite-FEN refines features—better align the activations with fine defect structures while background texture is suppressed; in (e), GRACE (Ours) combines both advantages, covering the core defect with the highest focus and strongest suppression of distractions. The contraction of the response region and the concentration of peaks provide an intuitive account of the model’s decision process when pursuing high Precision; under complex textures, the model more reliably highlights true defect areas, supporting the overall gains on small-defect detection. Consistent patterns are observed on crazing_299 and pitted_surface_279: hotspots shift from edge/background scatter in the baseline to clustered responses aligned with defect texture, with the combined model showing the best focus and background suppression.

## Discussion

This study targets steel-surface defects in NEU-DET and, building on the YOLO11s baseline, introduces two improvements: DS-CBSM++ and Lite-FEN. DS-CBSM++ uses gradient magnitude and predicted confidence as dual-modal signals; after log normalization, cardinality compensation, and bounded weighting, it establishes a stable dynamic sampling mechanism that focuses training on infrequent, low-confidence hard cases–improving learnability from the data-selection side. Lite-FEN injects lightweight channel- and spatial-path attention into the fine-scale P3 branch and employs residual scaling to progressively control strength, enhancing texture and boundary representations from the feature-space side with controllable computation. The two designs differ in focus yet are stackable: the former suppresses distribution bias and over-confidence, while the latter sharpens separability and local contrast.

Under the standard split and unified training protocol, the ablation outcomes are consistent with the Eigen-CAM visualizations^38^. With DS-CBSM++ alone, high-response regions concentrate around true defects and spurious background activations contract markedly. With Lite-FEN alone, fine and low-contrast textures receive more coherent boundary delineation, improving overall detection stability. The fused variant, GRACE (Ours), exhibits the most compact attention distribution and the most concentrated peaks; non-defect regions are effectively suppressed, yielding steadier discriminative purity and clearer localization. Class-wise, pitted_surface benefits most^39^, while rolled-in_scale and scratches show occasional fluctuations under complex textures, consistent with the slight conservatism of a strong denoising strategy for samples with extremely weak boundaries.

1. The precision–recall trade-off^40^: when the fused model emphasizes discriminative purity, recall on extremely low-contrast or blurred-boundary samples remains under pressure;
2. Scene generalization^41^: the data source is relatively homogeneous, and the proportion of hard cases differs from real production lines;
3. To better align with application needs and the data itself, we will: (i) perform lightweight deployment-side calibration by setting per-class score thresholds and simplifying NMS to reduce false alarms; (ii) remedy data gaps by collecting more production-line samples–especially extremely low-contrast, blurred-boundary, and complex-texture cases–and adjust sampling to match real prevalence; (iii) strengthen interpretability analysis by combining multi-layer responses to localize false positives/negatives, and accordingly fine-tune the weight bounds in DS-CBSM++ and the scaling strength in Lite-FEN.

The goal is to improve recall on extremely weak targets and cross-scene stability while keeping inference overhead minimal.

Under the unified setting, GRACE–compared with the YOLO11s baseline–raises mAP@0.5:0.95 from 42.66% to 43.66% (up 1.00 percentage points) and mAP@0.5 from 74.69% to 75.88% (up 1.19 percentage points); Precision is 73.62%, and Recall is 72.33%. These results are consistent with recent empirical trends in small-object modeling and long-tailed robust learning^42^. The parameter count is 9.56 M. Eigen-CAM visualizations show more concentrated attention with fewer spurious background activations, aligning with the metric gains; improvements are pronounced for crazing and pitted_surface, and detection of small-scale, low-contrast defects is more stable. The method is minimally invasive and deployment-friendly for online inspection; future work will validate cross-scene robustness using larger-scale production-line data and lightweight threshold calibration^43^. It should be emphasized that all experiments in this work are still conducted in an offline setting on public steel surface defect datasets, and GRACE has not yet been embedded into an online AOI system on a real production line for long-term validation. Integrating the proposed method into an actual industrial inspection pipeline and systematically evaluating its latency, stability and maintenance cost under real production constraints will be an important direction of our future collaboration with industrial partners.

## Methods

### Dataset and splits

The NEU-DET dataset contains 1,800 grayscale images of size $200\times 200$ pixels of steel-surface defects, covering six typical defect categories^44^. The dataset retains the original resolution without resampling; during training, images are uniformly scaled to $640\times 640$ and converted to the YOLO format^45^ via a custom script, transforming XML annotations into TXT labels and splitting the data at an 8:2 ratio. During conversion, a content-hash check found that patches_101.jpg and patches_105.jpg in the patches class are identical; to avoid duplicate counting we kept only patches_101.jpg, yielding 1,439 training images and 360 validation images. Although the per-class image count is balanced in NEU-DET (300 images per class), the number of annotated defect instances per class is long-tailed in our split.

Class- and size-wise statistics are as follows: *inclusion* and *patches* together account for approximately 46.13% of all instances, whereas pitted_surface is markedly underrepresented (approximately 10.35%). In total there are 4,186 instances: 447 small (10.68%), 2,772 medium (66.22%), and 967 large (23.10%). Object sizes follow the COCO convention^46,47^ at the $200\times 200$ resolution:

$$\text {small: area}<32^2,\quad \text {medium: }32^2\le \text {area}<96^2,\quad \text {large: }\text {area}\ge 96^2\ \text {pixel}^2.$$

Equivalently, in YOLO-normalized coordinates the thresholds are $(32/200)^2$ and $(96/200)^2$.In the remainder of this paper, we therefore refer to defect instances whose bounding-box area is smaller than $32^2\ \text {pixel}^2$ at the $200\times 200$ resolution (i.e., less than about 2.56% of the image area) as “small defects”, following the COCO convention, and use “medium” and “large” for the other two ranges. This distribution suggests that shallow textures and fine-grained edges are especially critical for detection.

In addition, to assess the cross-dataset generalization ability of GRACE, we further conduct experiments on two public steel surface defect datasets, GC10-DET and X-SDD. GC10-DET contains about 2,300 color images of hot-rolled steel strips annotated with ten typical surface defect categories; we follow the original 8/1/1 train/validation/test split and convert the annotations into the YOLO format. X-SDD is collected from continuous casting slabs; the detection version released on Roboflow Universe contains 2,258 images with seven defect classes and complex background textures. We adopt its official split of 1,850/269/139 images for train/val/test and resize all images to $640\times 640$ during training.

### Baseline model and implementation details

We adopt YOLO11s as the baseline detector. Compared with earlier anchor-based YOLOv5s variants, YOLO11s provides a modern anchor-free head and an enhanced feature pyramid while maintaining a similar parameter scale and real-time inference speed. Under the unified training setting used in this work, YOLO11s already achieves a strong mAP–F1 trade-off on NEU-DET (Table 1), so it serves as a representative and reasonably strong baseline for small-defect detection. In our setting, two factors are particularly relevant:

1. **Class-imbalance-induced training bias**: a standard uniform sampler tends to overfit high-frequency defects (e.g., inclusion and patches), while low-frequency categories (e.g., crazing) are under-represented^48^.
2. **Insufficient shallow features for small objects**: early-stage downsampling weakens fine texture and boundary cues, leading to missed detections of small or low-contrast targets^49,50^.

These limitations directly motivate the targeted improvements proposed in this work (DS-CBSM++ and Lite-FEN).

### Overall framework

We adopt YOLO11s as the detection baseline. The network is organized in a backbone–neck–head pipeline: the backbone performs hierarchical feature extraction; the neck, using FPN/PAN-style cross-scale fusion, aggregates multi-scale semantics; and the decoupled detection head outputs separate classification and bounding-box regression branches at three scales P3/P4/P5 (with strides of approximately 8/16/32)^51,52^.

**Fig. 4:**
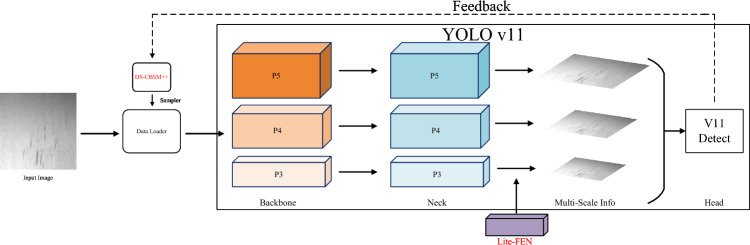
Schematic of the GRACE algorithm. Solid lines denote the forward-propagation path. DS-CBSM++ is located at the data-loading stage and receives confidence and gradient feedback from the detection head (dashed lines) to dynamically update the sampling strategy. Lite-FEN is inserted between the neck output and the detection-head input to enhance the P3 feature map.

To mitigate the sampling bias and insufficient shallow representations of YOLO11s in long-tailed and small-object scenarios, we construct the GRACE algorithm, keeping the backbone and detection head unchanged while adding two complementary components only on the training and feature sides: DS-CBSM++ and Lite-FEN. The former operates at the data-loading stage, fusing exponentially smoothed statistics of class cardinality, forward confidence, and backward gradients to produce normalized, boundary-clipped class sampling weights. The latter injects lightweight feature enhancement on the P3 path, reinforcing fine-grained textures and edges via a residual dual-path (channel and spatial) attention. Working in concert, the two modules improve small-object visibility and training stability under class imbalance with minimal additional inference overhead. The overall pipeline is illustrated in Fig. 4.

### DS-CBSM++: dynamic sampling with confidence-gradient balanced sampling mechanism

DS-CBSM++ is embedded at the data-loading stage and uses dual-signal feedback—gradient magnitude and predicted confidence—to generate class sampling weights, alleviating insufficient coverage of long-tailed categories. Both signals are smoothed with an exponential moving average (EMA); the design aligns with recent reweighting/resampling paradigms for imbalanced detection and incorporates density-aware reweighting^53^ to enhance stability.

First, we construct the base weights according to class frequencies (equation (6)):

6 $$\begin{aligned} \tilde{w}_c^{\textrm{base}} = \textrm{Normalize}\!\left( \frac{1}{\sqrt{f_c + \varepsilon }} \right) , \end{aligned}$$

where $f_c$ denotes the frequency of class $c$ in the training set; $\varepsilon$ is a numerical-stability term; $\textrm{Normalize}$ denotes mean-normalization across classes so that $\tfrac{1}{C}\sum _{c=1}^{C}\tilde{w}_c^{\textrm{base}}=1$.

To capture hard samples and suppress overconfident samples, we construct a dynamic adjustment term (equation (7)):

7 $$\begin{aligned} \Delta _{c} = \alpha \cdot \log \!\left( 1 + g_{c}^{\textrm{ema}}\right) + \beta \cdot \left( 1 - c_{c}^{\textrm{ema}}\right) , \end{aligned}$$

where $g_c^{\textrm{ema}}$ and $c_c^{\textrm{ema}}$ denote, respectively, the EMA of gradient magnitude and the EMA of confidence for class $c$. We fix $\alpha = 0.22$ and $\beta = 0.14$. The hyperparameters $\alpha , \beta$ are used to match the scales of $\log (1 + g_c^{\textrm{ema}})$ and $1 - c_c^{\textrm{ema}}$, and to suppress fluctuations of $\Delta _c$. Based on the observed magnitudes of class frequencies and warm-up EMA on NEU-DET ($\le 10$ epochs), exploratory ranges of $\alpha \in [0.20, 0.24]$ and $\beta \in [0.12, 0.16]$ yielded variations of mAP@0.5:0.95 and P/R within $\pm 0.5\%$; therefore, we fix the above values.

Building on the base weights, we derive the dynamic weights (equation (8)):

8 $$\begin{aligned} \tilde{w}_c^{\textrm{dyn}} = \operatorname {clip}\!\left( \operatorname {Normalize}\!\left( \tilde{w}_c^{\textrm{base}} \cdot \exp \!\left( \Delta _c\right) \right) ,\, w_{\min },\, w_{\max } \right) . \end{aligned}$$

We first perform cross-class normalization. After multiplying $\tilde{w}_c^{\textrm{base}}$ by $\exp (\Delta _c)$, we normalize again and then clip within $[0.85, 1.35]$ to suppress extreme amplification. This yields the dynamic weight $\tilde{w}_c^{\textrm{dyn}}$. We fix $w_{\min } = 0.85$ and $w_{\max } = 1.35$ throughout this work.

To stabilize early training and enhance discriminability in later stages, we apply epoch-wise progressive mixing to the final sampling weights (equation (9)–(10)):

9 $$\begin{aligned} & \gamma _e = \gamma _{\max }\cdot \min \!\left( 1, \frac{e + 1}{E_{\textrm{ramp}}}\right) , \end{aligned}$$

10 $$\begin{aligned} & w_c^{\textrm{final}} = (1 - \gamma _e)\cdot \tilde{w}_c^{\textrm{base}} + \gamma _e \cdot \tilde{w}_c^{\textrm{dyn}}, \end{aligned}$$

where $e$ is the epoch index starting from 0; $\gamma _e$ increases linearly from 0 to $\gamma _{\max }$ during the warm-up phase. Here, $\gamma _{\max }$ controls the maximum contribution of the dynamic term in the final sampling, and $E_{\textrm{ramp}}$ controls the duration of the linear ramp from warm-up to the steady stage. To balance early stability and later discriminability, we fix $\gamma _{\max } = 0.60$ and $E_{\textrm{ramp}} = 30$.

**Fig. 5:**
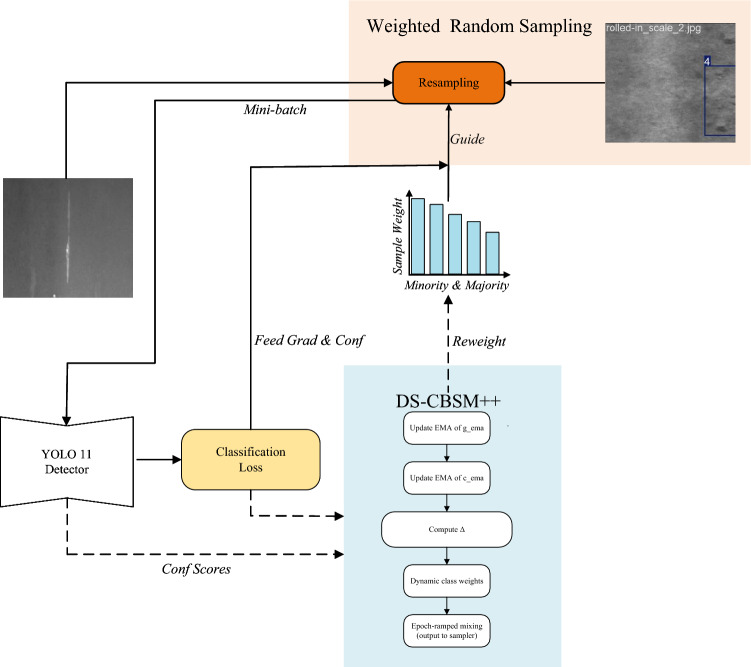
DS-CBSM++ workflow. Solid lines denote the forward data flow; input images are grouped into a mini-batch by a weighted random sampler (orange) and fed into the YOLO11 detector; dashed lines indicate the feedback loop: statistics of the classification loss (as a gradient surrogate) and the detection head’s sigmoid confidence are sent to the DS-CBSM++ module (blue) to update $g_c^{\textrm{ema}}$ and $c_c^{\textrm{ema}}$.

Implementation details and data flow are shown in Fig. 5. To suppress noise, only confidence scores $\ge 0.01$ are counted; every 20 mini-batches, a backward pass updates the EMA and the weight mapping. After the sampling weights are computed at the class level, they are mapped to the index set of samples belonging to that class to drive the next round of resampling.

### Lightweight feature enhancement network

**Fig. 6:**
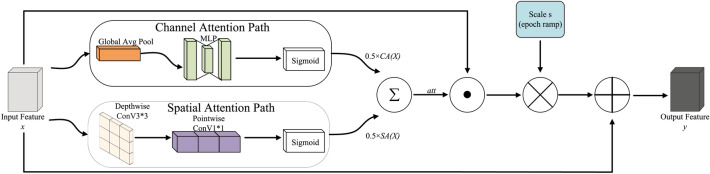
Lite-FEN architecture. The upper branch (channel-attention path) consists of GAP, MLP (channel squeeze/restore, compression ratio $r=4$), SiLU, and Sigmoid, producing $\textrm{CA}(x)$; the lower branch (spatial-attention path) consists of $\textrm{DWConv}\,3\times 3$, $\textrm{Conv}\,1\times 1$ ($C\rightarrow 1$), and $\textrm{Sigmoid}$, producing $\textrm{SA}(x)$. The two branches are fused with equal weights to obtain $\operatorname {att}(x)$, which is then used in a bounded residual enhancement.

Lite-FEN is injected at P3 to perform lightweight enhancement on the detail layer that is more sensitive to small objects^54^, as illustrated in Fig. 6. The module comprises two parallel paths—channel attention and spatial attention—whose outputs are fused with equal weights to form the attention map^55^, which is added to the input features in a bounded residual manner; no learnable fusion coefficients or additional normalization are introduced.

We fuse the channel-attention and spatial-attention paths with equal weights to obtain (equation (11)):

11 $$\begin{aligned} \operatorname {att}(x) = \tfrac{1}{2}\,\textrm{CA}(x) + \tfrac{1}{2}\,\textrm{SA}(x), \end{aligned}$$

where $x$ denotes the $P_3$ input feature. $\textrm{CA}(x)$ consists of $\textrm{GAP}$; $\textrm{Conv}\,1\!\times \!1$ (channels $C \rightarrow C/r$), $\textrm{SiLU}$, $\textrm{Conv}\,1\!\times \!1$ ($C/r \rightarrow C$), and $\textrm{Sigmoid}$ in sequence (compression ratio $r=4$); $\textrm{SA}(x)$ consists of $\textrm{DWConv}\,3\!\times \!3$, $\textrm{Conv}\,1\!\times \!1$, and $\textrm{Sigmoid}$, yielding a single-channel spatial weight that is broadcast along the channel dimension. Hence $\operatorname {att}(x) \in [0,1]^{H\times W \times C}$, and has the same shape as $x$.

To control the enhancement strength and stabilize early training, we set a linearly ramped residual ratio with epoch (equation (12)):

12 $$\begin{aligned} s_e = s_{\textrm{base}} \cdot \min \!\left( 1, \frac{e + 1}{E_{\textrm{ramp}}}\right) , \end{aligned}$$

and clip within $[s_{\min }, s_{\max }]$ (equation (13)):

13 $$\begin{aligned} \hat{s}_e = \operatorname {clip}\!\bigl (s_e, s_{\min }, s_{\max }\bigr ), \end{aligned}$$

where $s_{\textrm{base}}$ denotes the target strength of the residual; $s_{\min }$ and $s_{\max }$ globally bound the enhancement magnitude. To follow a “weak residual + upper-bound constraint” and to avoid early-training oscillation, we fix $s_{\textrm{base}} = 0.20$, $s_{\min } = 0.05$, and $s_{\max } = 0.30$ throughout all experiments.

The module output is (equation (14)):

14 $$\begin{aligned} y = x + \hat{s}_e \cdot \bigl (x \odot \operatorname {att}(x)\bigr ), \end{aligned}$$

$\odot$ denotes element-wise multiplication; $y$ is the output feature of Lite-FEN and is fed directly into the subsequent layers of the detection head.

Lite-FEN is inserted at the input of the detection head and operates only at $P_3$, $\hat{s}_e$ is updated each epoch by the trainer and is non-learnable. The module consists only of $1\times 1$ pointwise and $3\times 3$ depthwise convolutions together with element-wise operations; gradients flow only through the two attention branches and the backbone convolutions. The additional parameter count is small.

## Supplementary Information

Supplementary Information.

## Data Availability

The NEU-DET steel-surface defect dataset analysed in this study is publicly available; accessible download link: https://zenodo.org/records/16882077. The GC10-DET dataset analysed in this study is publicly available; an access page is provided at https://github.com/lvxiaoming2019/GC10-DET-Metallic-Surface-Defect-Datasets. The X-SDD data analysed in this study were obtained from a public Roboflow Universe object-detection dataset release (Project: https://universe.roboflow.com/yolov5-dh3rz/x-sdd). Processed artefacts supporting the findings (YOLO-format labels, train/val/test splits and figure source files) are available from the corresponding author on reasonable request.

## Code availability

The code used in this study will be made publicly available upon publication; the repository link/DOI will be added to the final article.
