# Supplementary material for: Confidence–gradient reweighting and lightweight feature enhancement algorithm for steel surface defect detection
Source: Sci Rep. 2026 Jan 18;16:5676. doi: 10.1038/s41598-026-36543-w (PMC12891620; doi:10.1038/s41598-026-36543-w)
Supplement: Supplementary file 1 — Supplementary Information. [file 41598_2026_36543_MOESM1_ESM.pdf]

Supplementary Information

Confidence–Gradient Reweighting and Lightweight Feature Enhancement Algorithm for Steel Surface Defect Detection

Authors
Linxuan Chen[1], Cunhan Guo[2], Xiaofang Wu[1], Huilin Xu[3], Shuangmei Chen[4], Junwu Lin[3]*

Affiliations
[1] College of Intelligent Manufacturing, Putian University, Putian 351100, China
[2] School of Computer Science and Technology, Beijing Institute of Technology, Beijing 100081, China
[3] College of Artificial Intelligence, Putian University, Putian 351100, China
[4] Fujian Putian Licheng Paper Industry Co., Ltd., Jiaoxi Village, Huating Town, Chengxiang District, Putian 351100, China

*Correspondence: ljw202128@ptu.edu.cn

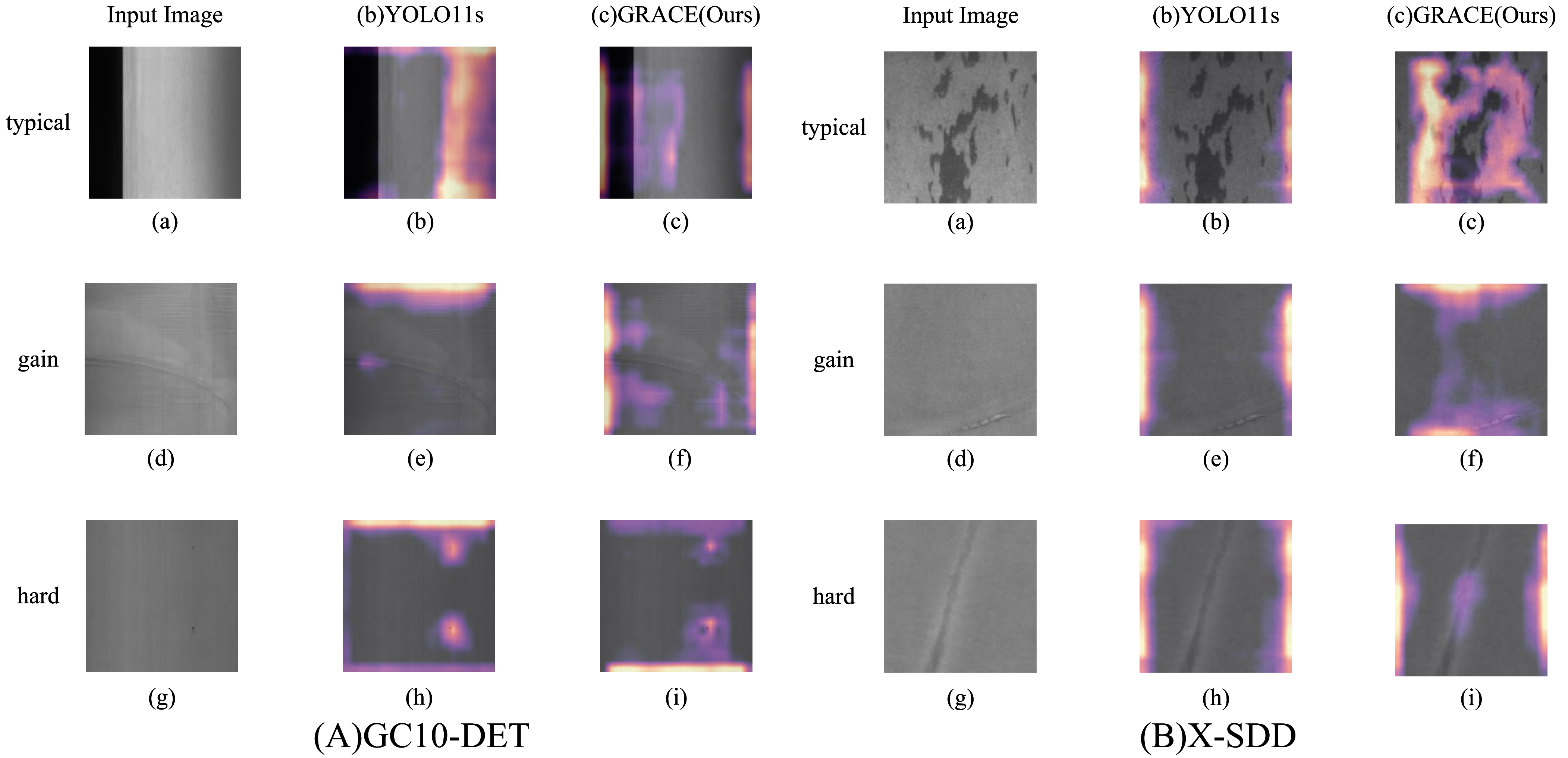

Supplementary Fig. S1 | Eigen-CAM visualizations on external datasets. Eigen-CAM heatmaps are shown for the baseline YOLO11s and GRACE on (A) GC10-DET and (B) X-SDD. For each dataset, three representative validation images are selected automatically using fixed and reproducible rules based on per-image detection performance: typical (both models correctly detect the defect and the performance is close to the dataset median), gain (the image with the largest recall improvement of GRACE over YOLO11s), and hard (the image with the lowest value of max(recall of YOLO11s, recall of GRACE), i.e., both models perform poorly). Columns show the input image and the corresponding Eigen-CAM heatmaps; higher-intensity regions indicate stronger feature activations.
